# The Influence of Affective Empathy and Autism Spectrum Traits on Empathic Accuracy

**DOI:** 10.1371/journal.pone.0098436

**Published:** 2014-06-06

**Authors:** Marije aan het Rot, Koen Hogenelst

**Affiliations:** Department of Psychology and School of Behavioral and Cognitive Neurosciences, University of Groningen, Groningen, Netherlands; French National Centre for Scientific Research, France

## Abstract

Autism spectrum disorder is characterized by interpersonal deficits and has been associated with limited cognitive empathy, which includes perspective taking, theory of mind, and empathic accuracy (EA). The capacity for affective empathy may also be impaired. In the present study we aimed to determine if EA in normally developing individuals with varying levels of autism spectrum traits is moderated by trait affective empathy. Fifty male and fifty female participants (‘perceivers’) completed the Autism-Spectrum Quotient and the Balanced Emotional Empathy Scale to assess autism spectrum traits and trait affective empathy, respectively. EA was assessed using a Dutch-language version of a previously developed task and involved rating the feelings of others (‘targets’) verbally recounting autobiographical emotional events. Targets varied in trait emotional expressivity, assessed using the Berkeley Expressivity Questionnaire. Perceivers with more autism spectrum traits performed worse on the EA task, particularly when their trait affective empathy was relatively low. Interpersonal deficits in autism spectrum disorder may be partially explained by low cognitive empathy. Further, they might be aggravated by a limited capacity for affective empathy.

## Introduction

The recently published DSM-5 includes diagnostic criteria for autism spectrum disorder (ASD), a neurodevelopmental disorder characterized by the presence of restricted, repetitive behaviors and persistent interpersonal deficits [1]. These symptoms and the underlying traits are thought to exist on a continuum. Individuals varying in autism spectrum traits may range from displaying normal psychosocial functioning to having severe problems in daily life. Autism spectrum traits are generally considered detrimental to daily functioning. However, Baron-Cohen [2] has argued that autism spectrum traits may be costly in some contexts but beneficial in other contexts. Specifically, individuals with these traits may have difficulties interacting with complex emotional beings (e.g., other humans) but exhibit great skill in analyzing abstract, technical, or organizable systems that are non-emotional (e.g. mathematics, machines, and mineral collections). Thus, one potential explanation for the repetitive behaviors and interpersonal deficits seen in ASD is that patients have extensive systemizing skills and at the same time relatively limited empathizing skills.

The ability to empathize with others is thought to encompass both affective and cognitive processes [3]–[5]. Affective empathy can be defined as the degree to which one can sense, or recognize implicitly, the feelings of others [6], [7]. When others experience difficult situations, sensing their feelings may elicit concern and compassion and subsequently motivate people to approach and provide support [8]. Thus, affective empathy can facilitate pro-social responses.

Cognitive empathy has been operationalized in terms of perspective taking [4], theory of mind [9], and empathic accuracy (EA) [10]. These abilities are more intellectual in nature and do not necessarily require sensing the feelings of others [8]. Nonetheless, cognitive empathy may be facilitated by concurrent affective empathic processes, such as physiological responses to social-emotional stimuli [11].

ASD has mostly been associated with deficits in cognitive empathy [2], [9], [12]. Several studies have assessed affective empathy in ASD, using subjective as well as more objective measures. Dziobek et al. [13] used the Multifaceted Empathy Test and found no significant differences between adults with ASD and controls on test components designed to assess affective empathy. Schwenck et al. [14] asked children to watch emotional video clips and indicate after each clip how much it had affected them. Children diagnosed with ASD provided ratings that were similar to those of a group of control children. Similarly, Deschamps et al. [15] asked children to complete a story task that involved labeling the affective state of the protagonist in the stories as well as their own and found that the affect reported by children with ASD matched that of the protagonist as often as did the affect reported by typically developing controls. Recently, Hadjikhani et al. [16] used functional magnetic resonance imaging to assess neurophysiological responses to observing other people in pain and found no significant differences between a group of adolescents and adults diagnosed with ASD and a group of controls in the activation of brain areas thought to be involved in shared pain experiences. These studies and others [17], [18] suggest affective empathy is intact in ASD.

Nevertheless, reports of limited affective empathy in ASD do exist [19]–[25]. For example, Sigman et al. [22] assessed behavioral responses to adults showing distress and found that, compared to controls, children with ASD showed less concern towards both their parents and the experimenters. Minio-Paluello et al. [24] assessed responses to observing other people in pain, using electromyography, and reported that adults with ASD show limited neurophysiological modulation of observed pain experiences. In a sample of adolescents and adults diagnosed with ASD, McIntosh et al. [25] found impairments on an electromyography paradigm involving automatic mimicry of facial expressions. Across these studies, it could be argued that general attention problems and other neuropsychological impairments explain the presented findings. However, Mathersul et al. [26] tested a large sample of adults with high-functioning ASD and controls carefully matched for neuropsychological function on several ecological measures. They also concluded that ASD is associated with limited affective empathy. Further, Mathersul et al. [27] have argued that affective empathy is highly variable within the ASD population. Thus, there is evidence that ASD may be characterized by poor cognitive empathy and poor affective empathy. Similarly, levels of autism spectrum traits in the general population may be negatively associated with both affective empathy and cognitive empathy.

Existing laboratory measures of perspective taking, theory of mind, and EA all assess how receivers of social-emotional information think about the senders of this information. Notably, during real-life interpersonal encounters, senders of social-emotional information are also the targets of any empathic responses elicited in the perceivers by the received information. Thus, the expressed feelings of senders play an important role in cognitive empathy and interpersonal dynamics. Nonetheless, only laboratory measures of EA include assessments of how senders actually feel [28], [29]. This greatly improves their ecological validity compared to that of laboratory measures of perspective taking and theory of mind. Laboratory measures of EA may even be used instead of a daily diary procedure, which by nature has very high ecological validity [29].

Zaki et al. [28] developed a laboratory measure of EA by creating a set of 40 video clips of 11 individuals (targets) verbally recounting emotional autobiographical events. After producing their personal clips, the targets watched these clips while providing continuous ratings to indicate how they felt when recounting their autobiographic events. Subsequently, 33 different individuals (perceivers) watched the clips and used the same continuous rating scale to indicate how they thought targets felt while recounting their autobiographical events. Zaki et al. [28] defined EA for each clip as the correlation between perceiver ratings and target ratings obtained for that clip. They found that perceivers attained higher levels of EA if they scored higher on a self-report measure of trait affective empathy, the Balanced Emotional Empathy Scale (BEES) [7], and were watching targets who scored relatively high on a self-report measure of trait emotional expressivity, the Berkeley Expressivity Questionnaire (BEQ) [30]. Thus, the ability to *feel* others’ feelings may facilitate *knowing* their feelings, but only if others express their feelings well enough. The task by Zaki et al. [28] can reveal context-specificity in (state) EA much better than more traditional (trait) measures of cognitive empathy. This and the use of ecologically valid stimuli are major advantages of the task.

Individuals with high-functioning ASD have previously been found to display poor EA on a similar task [31], [32]. Further, Bartz et al. [33] administered an abbreviated version of the task developed by Zaki et al. [28] to 27 men who also completed the Autism-Spectrum Quotient (AQ) [34]. The study revealed a negative association between EA and subclinical autism spectrum traits. More specifically, this negative association was observed after intranasal administration of a placebo but not after intranasal administration of oxytocin. The conclusion drawn from this study was that oxytocin, a neuropeptide, can improve EA in individuals with subclinical autism spectrum traits to the extent that they are indistinguishable from individuals without these traits.

As stated above, Zaki et al. [28] found that the trait emotional expressivity of the senders of social-emotional information (‘targets’) moderates the association between trait affective empathy and state EA in receivers of the information (‘perceivers’). Based on this finding, Bartz et al. [33] statistically controlled for target emotional expressivity. However, by controlling for target emotional expressivity it remained unclear whether the extent to which individuals with autism spectrum traits show reduced EA (and the extent to which they improve with oxytocin) is influenced by the degree to which others express their feelings. If others’ expressivity were to moderate the association between autism spectrum traits and reduced EA, then it might be possible to improve the empathic responses of individuals with autism spectrum traits by instructing people in their environment to be more explicit when communicating social-emotional information to the individuals with autism spectrum traits.

### The Present Study

There were three main aims to the present study. Primarily, we investigated the influence of autistic spectrum traits and trait affective empathy on EA in a Dutch version of the task developed by Zaki et al. [28]. More specifically, Aim 1 of our study was to extend the previous finding of a negative association between EA and autism spectrum traits [33]. On the one hand we did this by including both genders in our study. While Bartz et al. [33] restricted the study to male perceivers and did not consider the gender of the targets, for various reasons it seems relevant to explore the impact of both perceiver gender and target gender on EA: compared to women, men tend to be more likely to have autism spectrum traits [34], qualify for ASD [1], and score lower on the BEES [35] and the BEQ [30]. On the other hand we considered trait affective empathy as a potential moderator of the negative association between EA and autism spectrum traits. We did this because there is evidence that individuals with ASD show deficits in both cognitive empathy and affective empathy [26]. We were able to test our hypotheses for Aim 1 by conducting our study in a large sample of male and female perceivers.

Aim 2 was to replicate Zaki et al. [28], who found that EA was related to trait affective empathy when perceivers were rating targets with higher levels of emotional expressivity but not when perceivers were rating targets with lower levels of expressivity. Our version of the EA task previously developed by Zaki et al. [28] included a different set of targets, also with varying levels of emotional expressivity. Our hypothesis that target expressivity would moderate the link between perceiver affective empathy and perceiver cognitive empathy (i.e., EA) was directly based on the findings by Zaki et al. [28]. However, since affective empathy may facilitate cognitive empathy [11], but cognitive empathy does not necessarily require affective empathy [8], we also considered the possibility that perceiver EA would mostly be influenced by target emotional expressivity. Further, given gender differences in emotional expressivity and affective empathy [30], [35], in our analyses we considered the possible roles of perceiver gender and target gender. Furthermore, we used not only different targets than Zaki et al. [28], but also a larger sample of perceivers.

Aim 3 of the study pertained to a potential limitation of the EA task. The task was thought to have good ecological validity compared to other laboratory measures of cognitive empathy because (1) stimuli consist of video clips of targets verbally recounting autobiographical emotional events (rather than static pictures of facial expressions, for example), and (2) targets were asked to indicate how they felt while recounting their personal events. However, targets might not necessarily have been good at rating their feelings. They varied in trait emotional expressivity as assessed using the BEQ [30]. Since BEQ scores are relatively low in individuals who have difficulty describing their feelings, i.e. who have alexithymia [36], targets with lower BEQ scores may have had more difficulty indicating how they felt while recounting their personal events than targets with higher BEQ scores. Consequently, in previous studies [28], [33] target emotional expressivity may have been the primary factor influencing perceiver EA when target BEQ scores were high, but target emotional expressivity and unreliable target ratings may both have influenced perceiver EA when target BEQ scores were low. Thus, Aim 3 was to exclude the possibility that EA calculations from video clips of targets with low BEQ scores confound study results. For this reason, we repeated all analyses using for each video clip the mean rating of all perceivers for that clip, instead of the rating by the target in the clip.

## Materials and Methods

### Ethics Statement

We obtained approval to conduct the study from the Ethics Board of the Department of Psychology at the University of Groningen and obtained written informed consent from all participants as described under Procedure. We conducted the study in accordance with the Declaration of Helsinki.

### Participants

After obtaining study approval, we posted ads and handed out flyers in university buildings to recruit a group of individuals we will refer to as perceivers. The only exclusion criterion was having an insufficient knowledge of the Dutch language. We recruited 50 male and 50 female perceivers who were relatively homogeneous in age (*M* = 21.74 years, *SD* = 5.07) and educational background (94% were students). There were no significant differences between the genders on the demographic variables (Table 1). Participants received partial course credit or a minimal monetary remuneration for time spent in the study.

**Table 1:** Demographic and questionnaire data of the perceivers.

	Men (N = 50)	Women (N = 50)
**Demographic data**		
Age in years	22.46 (6.23)	21.02 (3.46)
Student	92%	96%
Bachelor degree or higher	14%	22%
Single	78%	52%
**Questionnaire data**		
Right-handedness	78%	86%
BEES score***	21.12 (22.71)	47.98 (22.27)
AQ score^†^	15.26 (7.00)	12.92 (5.55)
Questionnaire difficulty^a^*	2.06 (1.13)	1.66 (0.69)
Questionnaire accuracy^b^	5.10 (0.97)	5.38 (0.90)
**Empathic accuracy task data**		
Raw score *r* on all clips	0.57 (0.49)	0.57 (0.49)
Raw score *r* on negative clips	0.55 (0.44)	0.53 (0.45)
Raw score *r* on positive clips	0.59 (0.54)	0.60 (0.52)
Task difficulty^a^**	3.04 (1.26)	3.68 (1.10)
Task accuracy^b^	4.64 (0.90)	4.92 (0.90)

*Note*. ^†^p<0.10, *p<0.05, **p<0.01, ***p<0.001. AQ = Autism-Spectrum Quotient. BEES = Balanced Emotional Empathy Scale. ^a^Answer options 1 (not at all difficult) to 6 (extremely difficult). ^b^Answer options 1 (not at all accurate) to 6 (extremely accurate). Data in means (*SD*s) unless indicated otherwise.

(N = 100).

### Measures

To assess autism spectrum traits, we used a Dutch translation of the Autism-Spectrum Quotient (AQ) [34]. The AQ includes 50 self-report items rated on a 4-point Likert scale. Higher scores are indicative of having more autistic spectrum traits. Hoekstra et al. [37] previously determined the reliability and validity of the Dutch AQ.

To assess trait affective empathy, we used a Dutch translation of the Balanced Emotional Empathy Scale (BEES) [7]. The BEES includes 30 self-report items rated on a 9-point Likert scale, with 14 items reverse-scored. Higher BEES scores are indicative of higher subjective affective empathy. The Dutch BEES was generated for use in patients with traumatic brain injury by H.J. Evers, J.M. Spikman, and A.C. Visser-Keizer, using a translation/back-translation/adaptation method (personal communication). The Cronbach coefficient α in our study was 0.87, implying good reliability. Scores were higher in participants with fewer autism spectrum traits according to the AQ, *r*(100) = −0.26, *p*<0.009. This supports the construct validity of the Dutch BEES.

To measure EA, we developed a computer task similar to the one by Zaki et al. [28]. Prior to the present study, we generated a library of video clips from 6 men and 5 women. We invited these targets to the lab for a study on the display of emotions on video. Targets first completed a Dutch version of the Berkeley Expressivity Questionnaire (BEQ) [38], a 16-item self-report measure of trait emotional expressivity which includes the facets of impulse strength, negative expressivity, and positive expressivity. We obtained the Dutch BEQ from Swart et al. [36]; construct validity of the total and subscale scores can be derived from this study. We then asked targets to write down, on separate sheets of paper, the four most negative and the four most positive autobiographical events they were comfortable discussing. Targets subsequently recounted these events verbally, in random order, while being recorded on video. After each event, targets rated the overall valence and arousal of their emotions during the recording. Further, within 30 minutes after recounting all eight events, targets watched their personal video clips and used a dial with their right hand to continuously rate how they felt while describing each event. The rating dial anchors were 1 (extremely negative) and 9 (extremely positive) and corresponded with a 9-point Likert scale visible on the screen below the video. After targets had watched their personal clips, we debriefed them and asked for consent to use the clips as stimuli in future studies. The mean age of the five male and four female targets who provided consent was 33.33 years (*SD* = 14.15). Their mean BEQ total score was 4.53 (*SD* = 1.00). Male and female targets did not differ significantly in age, *t*(7) = 0.47, *p*>0.65, or BEQ total score, *t*(7) = −2.12, *p*>0.07. Considering the three BEQ facets, male targets reported less negative expressivity than female targets, *t*(7) = −3.09, *p*<0.02, but did not differ significantly on positive expressivity, *t*(7) = −2.14, *p*>0.06, and impulse strength, *t*(7) = −0.66, *p*>0.53.

For the computer task we discarded stimulus videos that the targets had rated low on arousal or that showed limited temporal variability in the continuous ratings. To enable future use of the task in studies with a repeated-measures design we generated two sets of 24 video clips, comparable in terms of the number of negative and positive events, the representation of male and female targets, clip length (*M* = 118.02 seconds, *SD* = 48.12), and overall arousal (*M* = 7.46, *SD* = 0.97) and valence (negative events: *M* = 2.25, *SD* = 1.03; positive events: *M* = 7.50, *SD* = 0.72). In each stimulus set the number of stimuli per target varied from one to three.

Targets generated more negative continuous ratings when watching the video recordings of personal events previously indicated as eliciting more negative valence, *r*(24) = 0.49, *p*<0.02, and more arousal, *r*(24) = 0.46, *p*<0.03. They also generated more positive continuous ratings when watching the video recordings of personal events previously indicated as eliciting more positive valence, *r*(24) = 0.52, *p*<0.009, and more arousal, *r*(24) = 0.40, *p*<0.06.

### Procedure

Upon arrival into the lab, we asked perceivers to read and discuss an information sheet and provide written informed consent. Perceivers then completed the BEES, the AQ, and the computer task, respectively. During the task perceivers watched one of the two stimulus sets and continuously rated how negative or positive they thought the target in each video recording felt while discussing an event. Each stimulus set was viewed by 25 male and 25 female perceivers. We randomized the order of the clips per perceiver, but there were never more than two positive or negative videos in a row, there were never more than two videos with a target of the same gender, and the same target never appeared more than twice in a row. Perceivers used the same rating dial as the targets and were also instructed to use it with their right hand. After each video they returned the dial to ‘neutral’.

Completion of the task took about 50 minutes. Afterwards perceivers completed a feedback form that asked about their difficulty with the task and with the questionnaires, and about their accuracy on both types of measures. Perceivers could also indicate if they had recognized any targets.

### Data Analyses

Seven perceivers recognized one or two targets. We discarded the data pertaining to these eight perceiver/target combinations. In addition, we discarded 25 perceiver/stimulus combinations because equipment failure sometimes resulted in an incomplete showing of a video clip. We averaged the remaining continuous rating data from both targets and perceivers across five-second periods. Visual inspection of targets’ and perceivers’ continuous ratings suggested that targets and perceivers would sometimes return the rating dial to ‘neutral’ just before the end of a video. Therefore we also discarded the final five seconds of all target and perceiver ratings.

Subsequent data steps were performed in SAS 9.3 for Windows (SAS, Cary, NC). We removed first-order autocorrelations from the continuous rating data of both targets and perceivers using the Yule-Walker method, after dropping the first five seconds of each video clip. This is equivalent to the Cochrane-Orcutt method used by Zaki et al. [28]. Per clip we then correlated perceiver ratings of targets’ feelings and target ratings of their own feelings. The resulting correlation coefficient *r* defined perceivers’ EA score for each video clip. The total number of EA scores was 2345 (100 perceivers each saw 24 target clips, minus excluded data as described above). These scores underwent a Fisher *z* transformation prior to further analysis.

We entered variables hypothesized to predict EA in mixed linear models. Variables included AQ score (continuous), BEES score (continuous), and Perceiver gender (male, female) at perceiver level and BEQ score (continuous), Target gender (male, female), and Valence (negative, positive) at target level. We standardized perceiver AQ and BEES scores and target BEQ scores to z scores and treated both perceivers and targets as random effects. Since the two stimulus sets generated similar levels of EA, *F*(1,98) = 0.80, *p*>0.37, we omitted the perceiver-level variable Set from our analyses.

For Aim 1 we first considered EA as a function of perceiver AQ scores. We then added target BEQ scores and the BEQ by AQ interaction to the model. We also explored the BEES by AQ interaction. For Aim 2 we first considered EA as a function of perceiver BEES scores, target BEQ scores, and their interaction. We then added perceiver gender and target gender to the model. For Aim 3 we recalculated all EA scores using the averaged continuous rating data from all perceivers (*N* = 50 per film clip) instead of the continuous rating data from the individual targets.

The set α was 0.05. We examined significant interactions by estimating simple intercepts and slopes for predictor scores that were 1 SD above the sample mean (“high”) or 1 SD below the sample mean (“low”) and testing the significance of the difference between the two slope estimates [39].

Our data will be freely available upon request.

## Results

The mean raw *r* between perceivers’ and targets’ ratings was 0.57 (*SD* = 0.49). There were no significant differences in EA between male and female perceivers (Table 1).

### Influence of Perceiver Autistic Spectrum Traits (Aim 1)

The mean AQ score was 14.09 (*SD* = 6.39). There was no significant gender difference (Table 1). Standardized AQ scores were found to negatively predict EA, *F*(1,98) = 7.37, *p*<0.008, *b* = −0.07, *d* = 0.55. Adding BEQ to the model did not meaningfully influence this finding and there was no significant AQ by BEQ interaction, *F*(1,2243) = 0.37, *p*>0.54. There was also no meaningful effect of adding Target gender to the model (instead of Target BEQ score). Further, there was no meaningful effect of adding Perceiver gender.

**Figure 1:**
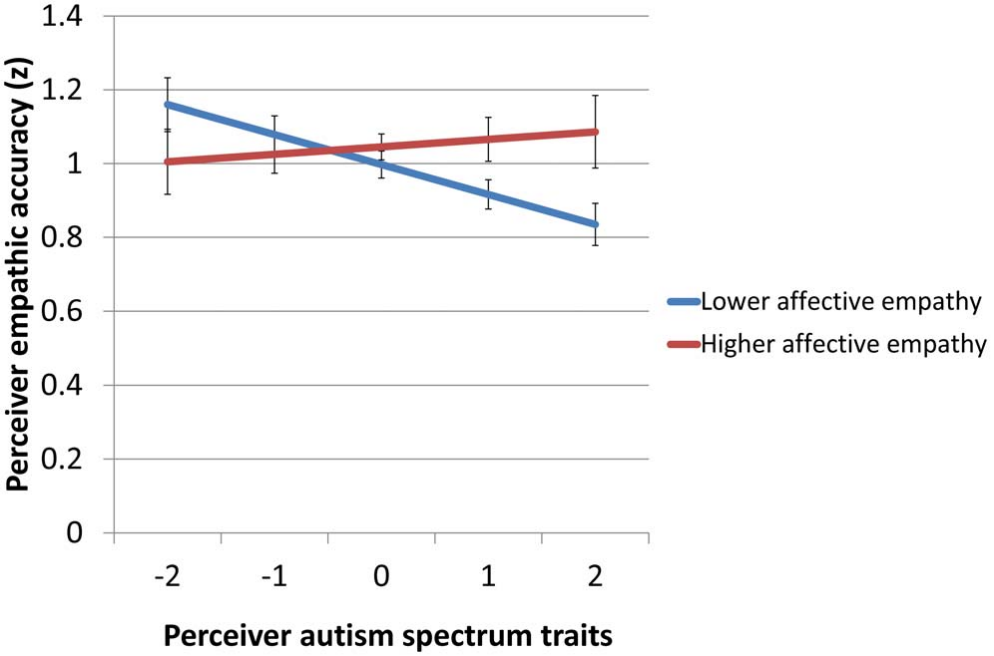
Empathic accuracy as a function of autism spectrum traits at higher and lower trait affective empathy. *Note:* Higher and lower affective empathy was defined as 1 SD above and below the mean BEES score, respectively. Perceiver autism spectrum traits are based on standardized AQ scores.

The mean BEES score was 34.55 (*SD* = 26.14). Female perceivers had higher BEES scores than male perceivers (Table 1). We then entered the BEES, AQ, and their interaction as predictors of EA. The AQ by BEES interaction was significant, *F*(1,96) = 5.31, *p*<0.03. Post-hoc probing of the interaction revealed a negative slope for AQ scores at lower BEES scores, *b* = −0.08, *t*(96) = −3.00, *p*<0.004, *d* = 0.61, and no significant association between AQ scores and EA at higher BEES scores, *b* = 0.02, *t*(96) = 0.47, *p*>0.64, *d* = 0.10. The difference between the two slopes was significant, *t*(96) = −2.31, *p*<0.03. Thus, autism spectrum traits negatively predicted EA in perceivers with lower affective empathy but not in perceivers with higher affective empathy (see Figure 1). BEQ scores, Target gender, and Perceiver gender did not moderate this finding.

### Effects of Perceiver Affective Empathy and Target Emotional Expressivity (Aim 2)

The BEES alone did not significantly predict EA, *F*(1,98) = 3.55, *p*>0.07, *b* = 0.05, *d* = 0.38. This did not change when we added target BEQ scores as a main effect, but the effect of the BEQ was significant, *F*(1,2244) = 221.20, *p*<0.0001, *b* = 0.32, *d* = 0.63. This did not change when we also added the BEQ by BEES interaction, and the interaction was not significant, *F*(1,2243) = 0.58, *p*>0.44.

**Figure 2:**
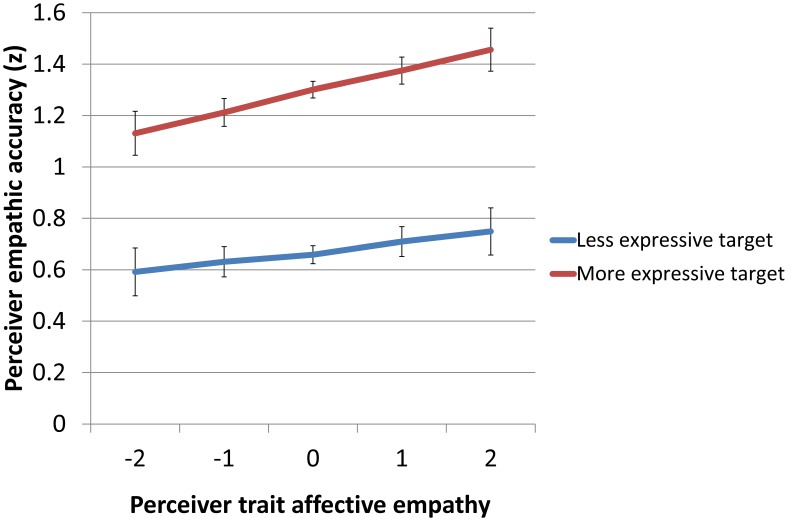
Perceiver empathic accuracy as a function of perceiver trait affective empathy for targets with higher or lower trait emotional expressivity. *Note:* Higher and lower target emotional expressivity was defined as 1 SD above and below the mean BEQ score, respectively. Perceiver trait affective empathy is based on standardized BEES scores.

Compared to male perceivers, female perceivers had similar EA scores but higher BEES scores (Table 1). We thus repeated the analyses described above with Perceiver gender added to the models as a potential moderator. The main effect of the BEES on EA was now significant, *F*(1,96) = 4.58, *p*<0.04, *b* = 0.06, *d* = 0.44. The main effect of BEQ remained significant, *F*(1,2243) = 221.11, *p*<0.0001, *b* = 0.32, *d* = 0.63. The BEQ by BEES interaction was not significant, *F*(1,2241) = 0.51, *p*>0.47. Thus, when taking perceiver gender into account, higher perceiver EA was predicted by a combination of higher perceiver affective empathy and higher target emotional expressivity (Figure 2).

Overall, our results were similar to those of Zaki et al. [28] in that (1) perceiver EA was highest when perceiver affective empathy and target emotional expressivity were both high, and (2) target emotional expressivity had a large effect on perceiver EA. However, our results were different in that the effects of perceiver affective empathy and target emotional expressivity on perceiver EA did not amplify each other.

Unlike in the study by Zaki et al. [28], perceiver EA differed with the valence of the video clips, with negative clips generating lower EA scores, *F*(1,99) = 49.72, *p*<0.0001, *d* = 1.42. Thus, in an additional set of analyses, we checked whether our results remained when taking the valence of the video clips into account. We found that valence did not moderate the independent effects of BEES and BEQ on EA.

Compared to female targets, male targets had lower BEQ negative expressivity scores (see Measures) and generated lower EA scores in perceivers, *F*(1,99) = 31.53, *p*<0.0001, *d* = 1.13. Thus, we also repeated all analyses using Target gender instead of the BEQ. Effectively the results did not change. When taking perceiver gender into account, higher perceiver EA was predicted by a combination of higher perceiver affective empathy and the target being female rather than male, but there was no significant interaction between these two predictors.

### Testing the Reliability of Target Ratings (Aim 3)

We repeated the analyses for Aims 1 and 2 using recalculated “EA” scores (see Data analyses for details). The mean raw *r* between averaged perceivers’ and individual perceiver ratings was 0.73 (*SD* = 0.42).

For Aim 1, the AQ by BEES interaction was not significant, *F*(1,96) = 3.54, *p*>0.06. Only a main effect of AQ scores on “EA” remained, *F*(1,98) = 3.98, *p*<0.05, *b* = −0.07, *d* = 0.40. Thus, the results changed somewhat when “EA” scores were calculated using the average of all perceivers’ continuous ratings instead of individual target ratings. For Aim 2, after including Perceiver gender in the model, there were main effects of the BEES, *F*(1,96) = 6.65, *p*<0.02, *b* = 0.10, *d* = 0.53, and the BEQ, *F*(1,2241) = 51.24, *p*<0.0001, *d* = 0.30, and the BEES by BEQ interaction was not significant, *F*(1,2241) = 0.25, *p*>0.61. We obtained similar results when we replaced the BEQ by Target gender.

In all, regardless of the way we calculated EA scores, we replicated the negative relation between autism spectrum traits and EA reported by Bartz et al. [33] but not the interaction effect of perceiver affective empathy and target emotional expressivity reported by Zaki et al. [28].

## Discussion

In the present study we aimed to extend previous results by Bartz et al. [33]. They reported a negative association between EA and autism spectrum traits in normally developing men. Our results replicated this finding in both female and male perceivers, regardless of whether they were rating female or male targets. Further, expressivity of the targets did not moderate the observed association between EA and autism spectrum traits.

Additionally, we observed a negative association between EA and autism spectrum traits among perceivers with lower self-reported trait affective empathy but not among perceivers with higher trait affective empathy. This corroborates previous findings of impairment on both affective and cognitive empathy measures in high-functioning individuals with ASD [19], [20], [26]. Other studies on empathy and ASD have not found significant differences between ASD individuals and controls in both the affective and the cognitive domain. A study comparing adults with ASD to controls matched for their level of alexithymia may help explain why only some ASD studies to date have found significant impairment in both affective and cognitive empathy [40]. The authors found no significant group difference in the ability to interpret others’ emotional states, however there was a negative association between participants’ task performance and their scores on an alexithymia questionnaire. Thus, differences in alexithymia among individuals with ASD may help explain why some studies have found impairments in affective empathy and others have not. This idea fits with our finding that the link between autism spectrum traits and EA depends on trait affective empathy. In a future study, to further elucidate this link, we will assess alexithymia.

In the study by Bartz et al. [33], there was a negative association between EA and autism spectrum traits among perceivers treated with a placebo but not among perceivers treated with oxytocin. Might oxytocin improve EA by increasing affective empathy? To date, studies designed to answer this question have produced mixed findings [41], [42]. However, oxytocin’s effects on pro-social behavior in organisms that are evolutionarily older than humans [43] are presumably better explained by effects on affective empathy than by effects on cognitive empathy. Moreover, in humans, assessing affective empathy without the influence of cognitive empathy is difficult with self-report measures. These measures include the BEES used in the present study. Electrophysiological measures could reveal information on the implicit rather than explicit experience of affective empathy in humans [11]. Thus, future studies should explore whether oxytocin-induced effects on cognitive empathy (including EA) are mediated by effects on (explicit and implicit) affective empathy.

We developed a Dutch-language equivalent of the EA task described by Zaki et al. [28]. A second aim of our study was to replicate their finding that the impact of trait affective empathy on state EA in receivers of social-emotional information (perceivers) is moderated by the emotional expressivity of the senders (targets). Instead we found that perceiver affective empathy (i.e. higher BEES scores) and target emotional expressivity (i.e. higher BEQ scores) both contributed independently to perceiver EA, with target emotional expressivity contributing substantially more than perceiver affective empathy (see reported effect sizes and Figure 2). It appears from both the present study and a previous one [28] that empathic accuracy is optimal when senders are good at expressing their feelings and receivers are good at sensing these feelings, but even when senders do not express their feelings well, for the correct labelling of these feelings receivers may still benefit from being good at sensing others’ feelings.

There were few differences between the task developed by Zaki et al. [28] and our task in terms of the number, average age, and gender distribution of the targets. Further, the targets selected for both studies were similar in terms of their BEQ total scores (*p*>0.42, J. Zaki, personal communication). Thus, between-study differences at target level are unlikely to explain the differences in results. Nevertheless, between-study differences at perceiver level may play a role. For example, the average BEES score in our study sample appeared lower than the average score found in other samples of undergraduate students [7], [44]. This might help explain why, in our study, perceiver affective empathy contributed substantially less to EA than target emotional expressivity. We also note that our sample was three times larger than the sample studied by Zaki et al. [28] and therefore less likely to yield a type I error in the data analyses.

The third and final aim of our study was to check whether EA scores may be confounded by targets with lower BEQ scores, and thus possibly higher levels of alexithymia [36]. In the computer task, EA is normally calculated per video clip and defined as the correlation between a target’s continuous ratings of his or her personal feelings and a perceiver’s continuous ratings of these feelings. To check the validity of this measure, “EA” was calculated using the correlation of each perceiver’s ratings for a clip to the average of all perceiver ratings for that clip. This way we effectively bypassed potentially unreliable targets. Targets with lower BEQ scores also generated lower “EA” scores, and the results obtained under aims 1 and 2 were largely replicated. Thus, while inexpressive targets may have confounded the EA data somewhat, this effect was small and did not alter the results in a meaningful way.

In conclusion, we showed that both men and women exhibit poorer EA when they have more autism spectrum traits. However, this may only be true for individuals who have both more autism spectrum traits and less trait affective empathy (Figure 1). This finding is potentially relevant to the social interactions of individuals with ASD and also provides support for the validity of the Dutch-language EA task. Future studies might test performance on this task in individuals with AQ scores in the clinical range, since these individuals perform poorly on other laboratory measures of EA [31], [32]. We recommend the additional assessment of affective empathy, either by self-report questionnaire [7] or by means of a physiological measure [11].
